# Effect on birth outcomes of a formalised approach to care in hospital labour assessment units: international, randomised controlled trial

**DOI:** 10.1136/bmj.a1021

**Published:** 2008-08-28

**Authors:** Ellen D Hodnett, Robyn Stremler, Andrew R Willan, Julie A Weston, Nancy K Lowe, Kathleen R Simpson, William D Fraser, Amiram Gafni

**Affiliations:** 1 Lawrence S Bloomberg Faculty of Nursing, University of Toronto, 155 College St, Toronto, ON, Canada, M5T 1P8; 2 Program in Child Health Evaluative Sciences, and Research Institute at SickKids, SickKids Hospital, Toronto, Canada; 3 Dalla Lana School of Public Health, University of Toronto, 155 College St, Toronto, ON, Canada, M5T 3M7; 4 University of Colorado Health Sciences Center, School of Nursing, Denver, CO, USA; 5 Labor and Delivery, St John’s Mercy Medical Center, St Louis, MO, USA; 6 Université de Montréal, Département d’obstétrique-gynécologie, Pavillon Roger-Gaudry, Montréal, QC, Canada; 7 Department of Clinical Epidemiology and Biostatistics, and Centre for Health Economics and Policy Evaluation, McMaster University, Hamilton, ON, Canada

## Abstract

**Objective** To determine if a complex nursing and midwifery intervention in hospital labour assessment units would increase the likelihood of spontaneous vaginal birth and improve other maternal and neonatal outcomes.

**Design** Multicentre, randomised controlled trial with prognostic stratification by hospital.

**Setting** 20 North American and UK hospitals.

**Participants** 5002 nulliparous women experiencing contractions but not in active labour; 2501 were allocated to structured care and 2501 to usual care.

**Interventions** Usual nursing or midwifery care or a minimum of one hour of care by a nurse or midwife trained in structured care, consisting of a formalised approach to assessment of and interventions for maternal emotional state, pain, and fetal position.

**Main outcome measures** Primary outcome was spontaneous vaginal birth. Other outcomes included intrapartum interventions, women’s views of their care, and indicators of maternal and fetal health during hospital stay and 6-8 weeks after discharge.

**Results** Outcome data were obtained for 4996 women. The rate of spontaneous vaginal birth was 64.0% (n=1597) in the structured care group and 61.3% (n=1533) in the usual care group (odds ratio 1.12, 95% confidence interval 0.96 to 1.27). Fewer women allocated to structured care (n=403, 19.5%) rated staff helpfulness as less than very helpful than those allocated to usual care (n=544, 26.4%); odds ratio 0.67, 98.75% confidence interval 0.50 to 0.85. Fewer women allocated to structured care (n=233, 11.3%) were disappointed with the amount of attention received from staff than those allocated to usual care (n=407, 19.7%); odds ratio 0.51, 98.75% confidence interval 0.32 to 0.70. None of the other results met prespecified levels of statistical significance.

**Conclusion** A structured approach to care in hospital labour assessment units increased satisfaction with care and was suggestive of a modest increase in the likelihood of spontaneous vaginal birth. Further study to strengthen the intervention is warranted.

**Trial registration** Current Controlled Trials ISRCTN16315180.

## Introduction

Since the late 1980s labour assessment units have become a routine feature in North American hospitals, but they remain uncommon elsewhere. The original intent of such units was to restrict hospital admission to those who were judged to be in active labour or experiencing complications, in accordance with the tenets of “active management of labour.”^1^ Care providers may pay little attention to variations in length of latent phase labour because of widespread beliefs that they are not associated with increased obstetrical risk.^2^ Latent phase labour may, however, involve painful contractions that occur intermittently for hours or days, and a prolonged latent phase may not be benign. Over 30 years ago the National Collaborative Perinatal Project reported an association between a prolonged latent phase and increased neonatal mortality and morbidity.^3^ A 1993 California study of more than 10 000 term deliveries of babies at no previous risk found that a prolonged latent phase (defined as >12 hours for nulliparous women and >6 hours for multiparous women) was independently associated with a higher risk of abnormalities during labour, caesarean delivery, low Apgar score, and need for neonatal resuscitation.^4^ Although many factors may contribute to a prolonged latent phase, two problems consistently associated with it are high maternal anxiety and a malpositioned fetal head.^5^ ^6^ ^7^ ^8^ ^9^ Thus the labour assessment unit offers an opportunity for both primary and secondary prevention of complications.

Several studies have found associations between anxiety before active labour and intrapartum complications.^6^ ^10^ ^11^ ^12^ Women in latent labour who expressed negative feelings about their ability to cope or had high pain ratings were more likely to develop intrapartum complications.^5^ Reframing negative thoughts to positive ones may reduce anxiety and the likelihood of subsequent complications. Brief cognitive restructuring interventions are logical extensions of the explanations and reassurance that are part of routine clinical practice in outpatient settings and have shown efficacy in reducing anxiety and pain.^13^ ^14^

Malposition of the fetal head has been associated with a prolonged latent phase, increased pain, higher maternal anxiety, complications in active labour, and higher rates of operative delivery.^15^ ^16^ ^17^ ^18^ Medical interventions during active labour, such as amniotomy, oxytocin, and epidural analgesia can be effective treatments for the problems resulting from malposition, but they do not increase the likelihood that the fetal head will rotate to the occiput anterior position.^19^ Simple positioning techniques may encourage rotation and descent.^15^

Caesarean delivery rates continue to increase.^20^ Although there has been some debate about whether women have the right to choose elective caesarean delivery, spontaneous vaginal birth is widely regarded as the safest method of birth for healthy mothers and babies at low risk.^21^ Controlled evaluations of intrapartum interventions have focused on active labour and have had little effect on the likelihood of caesarean delivery.^22^ ^23^ Using a pragmatic design we determined the effects on the likelihood of spontaneous vaginal birth of a formalised approach to care in labour assessment units, which focused on assessment of and anticipatory guidance on cognitive-emotional state, pain, and fetal position.

## Methods

The study was a multicentre, randomised controlled trial with prognostic stratification by hospital. Before the trial a group of nurses or midwives at each hospital were trained in the structured approach, with the number trained at each site reflecting the projected rate of enrolment. Additional training sessions were held during the recruitment phase in sites with substantial staff turnover. In the North American hospitals the two day training programme was carried out by nurse experts who worked in an organisation providing continuing education to labour nurses in Ontario, Canada. In the UK hospitals the training programme was carried out by an expert midwife instructor after consultation with the Canadian trainers, using an adaptation of the North American curriculum. At the end of the workshop each participant was given a manual, which reiterated course content and provided opportunities for the techniques to be practised before the onset of the trial. The box details the topics in the training programme, and the accompanying manual covered the components of structured care.

**Components of structured care**

The following components were taught in the training programme and used in the structured care group*

Normalise the environment—if the labour assessment unit is a clinical environment or offers little privacy, move to a lounge or empty room away from medical equipment; encourage use of comforting objects and spontaneous self comforting behaviours
Palpate to assess fetal position
Encourage maternal positions that promote fetal head rotation or relieve pain—standing and leaning forward; asymmetrical upright (standing, kneeling, sitting); sitting upright; sitting, leaning forward with support; kneeling, leaning forward with support; on hands and knees; side lying positions (pure side lying and exaggerated Sims or semiprone); abdominal lifting
Assess labour pain, both contraction pain and backache; assess physiological and behavioural indicators of pain; assess pain using a visual analogue scale
Demonstrate cognitive, behavioural, and sensory interventions to manage labour pain; be present continuously; encourage comfort measures, including breathing and relaxation, application of heat and cold, massage, shower or bath, movement; encourage visualisation techniques, including recalling past experiences when contentment has been felt, picturing the results of the contractions (cervical dilation, fetal rotation, and descent), and visualising each contraction as a mountain and each breath as a step towards the peak; help create a ritual behaviour to use with each contraction; suggest music and rhythmic techniques
Assess maternal emotional status; be aware of behavioural indicators of emotional distress; know how and when to ask direct questions, such as “What was going through your mind during that last contraction?” to identify coping related responses compared with distress related responses
Use interventions to reduce emotional distress: offer helpful replies to distress related responses; reframe negative thoughts to positive ones; discuss the importance of relaxation, rhythm, and ritual; scan the body for areas of tension to focus relaxation efforts
If hospital admission is not planned, discuss the importance of carrying on normal activities of daily living, with specific suggestions appropriate to time of day; offer anticipatory guidance about coping with labour as it becomes more active

^*^Content on positioning, interventions for pain, and assessment and interventions for emotional status was adapted from Simkin and Ancheta 1999^24^

Throughout the trial providers of structured care could communicate with the trainers and with each other on an electronic listserv. Before the launch of the trial at each centre the principal investigator (EH) held meetings with the staff at which she emphasised the uncertainty about the value of structured care and the importance of staff continuing to provide usual care to the control group, to maintain distinctions between both groups.

### Eligibility criteria

To be eligible to participate the hospitals had to have a pre-existing, geographically distinct labour assessment unit and a spontaneous vaginal birth rate of 75% or less. In all study sites the labour assessment units were the entry points for women who thought they were in labour. In North America the units were staffed by nurses and in the UK by midwives, but the approach to care was the same—that is, the purpose was to determine whether a woman should be admitted to the labour ward or sent home to await active labour. Women were eligible for the study if they were nulliparous, had a live singleton fetus in the cephalic position, had no contraindications to labour, were competent to give informed consent or had a parent or guardian who was competent to give informed consent, and were experiencing contractions but did not meet labour ward criteria for admission. Women were not eligible if the gestational age was less than 34 weeks, an induction of labour or caesarean delivery was planned, they had complications that necessitated hospital admission, they were likely to be transferred to the labour ward within one hour, or they had a doula or midwife providing continuous support.

### Treatment protocol

After basic assessment of labour (duration and frequency of contractions, status of membranes, status of the fetal heart rate, and assessment of cervical dilation as per hospital protocol) the women were assessed for trial eligibility. Baseline data were obtained after eligible women gave written, informed consent to participate and before randomisation. Determination of race or ethnicity was a combination of self assignment and observer assignment. Randomisation was centrally controlled and concealed, using an internet based service. The nurse or midwife accessed the trial website to obtain the participant’s study group allocation.

Immediately after randomisation women assigned to the experimental group received one to one care by a nurse or midwife trained in structured care. The structured care provider palpated the participant’s abdomen to assess fetal position and asked her to describe her thoughts during the last contraction and to rate pain intensity on a visual analogue scale. The provider used positioning techniques, comfort measures, and simple cognitive restructuring techniques such as positive visual imagery and reframing negative thoughts, and offered anticipatory guidance about coping with active labour (see box). If the woman was sent home without having delivered and if circumstances permitted, the provider was to telephone her later to check on her progress. When the woman returned, efforts were made to repeat structured care.

Women assigned to the control group received care by a nurse or midwife who had not been trained in structured care. One nurse or midwife often provided care to more than one woman. Usual care depended on many factors, including the provider’s workload, the provider’s knowledge of and beliefs about assessments of fetal position and cognitive-emotional state, and familiarity with appropriate interventions. Women who were sent home were advised to telephone the unit if they had any questions or concerns.

The length of time women received structured care or usual care was designed to reflect the usual time spent by women in labour assessment units (1-4 hours). In both groups the decision on whether to admit women to the labour ward or to send them home was made as per usual hospital policy. Labour assessment units offer varying degrees of privacy and in some settings it was possible that a patient randomised to the control group could overhear or witness parts of the care to a woman in the experimental group. Therefore no woman was invited to participate while a trial participant was in the labour assessment unit unless complete privacy could be assured. Only the nature of the nursing or midwifery care in the labour assessment unit varied between groups; all other care in the labour assessment unit and labour ward was in accordance with usual hospital practices and policies.

### Compliance

Centres were instructed not to randomise women unless providers of both usual care and structured care were available. Structured care providers could care for non-study women but not for women in the control group. Usual care providers could not care for women in the structured care group. Compliance was assessed in two ways. We expected that over 90% in each group would receive their assigned method of care (care from a provider trained or not trained in structured care) immediately after randomisation. In addition, providers’ reports of their care for women in the structured care group provided evidence of adherence to the main components of the intervention.

### Outcomes

The primary outcome was spontaneous vaginal birth. Secondary outcomes were the number of women who had no intrapartum analgesia or anaesthesia, had perineal trauma requiring suturing, and reported negative views of their care (a subsequent paper will report on costs). Other study outcomes included the number of women with more than two visits for assessment of labour; use of intrapartum oxytocics, regional analgesia, and electronic fetal heart rate monitoring; length of hospital stay; and indicators of short term and longer term maternal and neonatal morbidity that have been linked in previous research to method of delivery, including postnatal emotional distress, readmission to hospital of mother or baby for delivery related complications in the first 6-8 weeks after birth, neonatal transfer to a special care baby unit, and intrapartum fetal death or neonatal death.

Trained research nurses or midwives at each hospital abstracted relevant data from the medical records and entered the data into study forms on the trial website. Because the data collection included a question about compliance (whether a provider with training in structured care had cared for the participant), the research staff may not have been fully blinded during retrieval of data from the medical records. The primary and other medical outcomes were, however, objective data that were recorded in the medical records as part of routine practice. Audits of medical records at selected sites during the enrolment period showed neither systematic errors nor important random errors. Attending doctors were not explicitly informed of their patients’ group assignments and were rarely present in labour assessment units.

Participants were asked to complete a questionnaire 6-8 weeks after the birth, which focused on their health, their baby’s health, and their satisfaction with care. The questionnaire included the Edinburgh postnatal depression scale; a score greater than 12 is a reliable indicator of postnatal depressive symptomatology.^25^ Measurement of satisfaction is problematic because of the “what is, must be best” phenomenon.^26^ ^27^ A systematic review identified key factors influencing satisfaction with childbirth,^27^ and the questionnaire items were adapted from one of the most reliable and well validated population based surveys of satisfaction with childbirth.^28^ Participants could complete their questionnaires directly on the secure trial website,^29^ by post, or by telephone with the centre research nurse or midwife.

### Sample size calculation

During development of the trial protocol 10 hospitals provided the rates of spontaneous vaginal birth for women who would meet trial eligibility criteria. Rates ranged from 42% to 76% (mean rate 58%). In selecting the clinically important difference for the current trial we wanted to detect a 4% absolute difference in spontaneous vaginal birth rate—for example, eliminating one operative delivery for every 25 women treated. With a two sided α<0.05 and α=0.20 in hospitals at the extremes of the range, and when using the mean rate and taking the most conservative approach, we needed a sample size of 4932. The target sample size was 5000.

### Statistical analysis

We analysed the results according to intention to treat. For the primary outcome we used a significance level of 0.05 (two tailed). We set the significance level for secondary outcomes at 0.0125 and for other study outcomes at 0.005. Because we expected variation owing to the effects of unknown characteristics of the hospitals, the analytical approach allowed the proportion of women experiencing spontaneous vaginal birth and treatment effects to vary between hospitals. For binary outcome variables we compared the groups using a logistic regression model with a random hospital effect for the intercept and slope. We present the odds ratios and accompanying confidence intervals (corresponding to the preset P values for primary, secondary, and “other” outcomes). We used a similar logistic regression model to explore the interaction effects between baseline variables and treatment group on the primary outcome. For length of hospital stay we analysed data using a linear regression model with a random hospital effect for the intercept and slope, using the log of length of stay as the dependent variable. Statistical procedures were done using SAS version 9.1. For ratings of women’s views of their care we followed the standard practice of comparing the frequencies with which less than very positive views were reported.^26^ ^27^ ^28^

## Results

Twenty tertiary care teaching and community hospitals participated in the trial, eight in Canada, 10 in the United States, and two in the UK. Training in structured care was provided to 505 nurses and midwives; the remaining 1351 were available to provide usual care. The annual birth census at the hospitals ranged from 1200 to 9841. Although the labour assessment units were separate from the labour wards, they varied in design, size, and staffing, from multibed rooms with beds separated by curtains, which were staffed on a rotating basis by nurses or midwives, to private rooms in units with staff who worked only in the labour assessment unit.

We enrolled 5002 women between 1 May 2003 and 6 March 2007 (figure[Fig fig1]). For reasons of cost and feasibility, data were not collected on eligible women who did not participate in the trial. Centre staff, however, stated that women rarely refused to participate; those who did stated that they had been informed of the study just before being sent home from the labour assessment unit and did not want to delay their departure. The main reasons for failure to enrol eligible women were logistical, including staff workload, unavailability of a provider for structured care, or the presence in the unit of a trial participant.

**Figure fig1:**
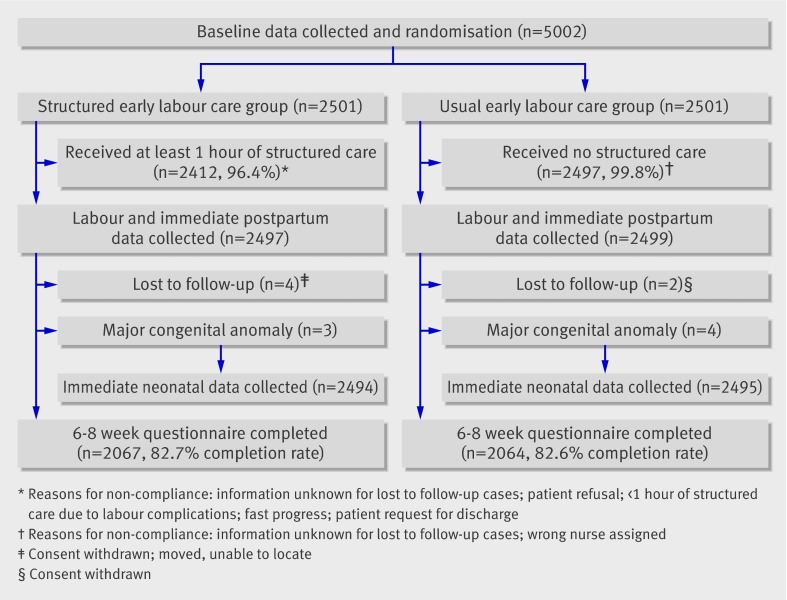
Flow diagram for trial

Table 1 shows the baseline characteristics of the sample. Immediately after randomisation the appropriate form of care was provided to 2412 of 2501 women (96.6%) in the structured care group and to 2497 of 2501 women (99.8%) in the usual care group. Structured care providers completed forms describing their activities for 2406 of the 2497 women in the structured care group (96.4%). The forms provided evidence of the consistency with which the components of the intervention were provided. For example, in the first episode of structured care, fetal position, maternal emotional state, and intensity of pain were assessed in over 99% (n=2402) of women in the structured care group. One or more structured care interventions were provided to all but seven women (99.8%).

**Table 1 tbl1:** Baseline characteristics of women in structured care and usual care groups. Values are numbers (percentages) unless stated otherwise

Characteristic	Structured care (n=2501)	Usual care (n=2501)
Mean (SD, minimum-maximum) age (years)	26.7 (5.87, 14-45)	26.6 (5.95, 15-44)
Race or ethnicity:		
White	1693 (67.7)	1704 (68.1)
Black	197 (7.9)	202 (8.1)
Latin American	264 (10.6)	257(10.3)
Chinese	63 (2.5)	62 (2.5)
Japanese	6 (0.2)	5 (0.2)
Korean	12 (0.5)	11 (0.4)
South and South East Asian	191 (7.6)	174 (7.0)
West Asian	58 (2.3)	58 (2.3)
Aboriginal	9 (0.4)	21 (0.8)
More than one race	7 (0.3)	5 (0.2)
Unknown	1 (0.04)	2 (0.1)
Education:		
Lower than high school	263 (10.5)	268 (10.7)
Completed high school	793 (31.7)	799 (32.0)
Post-secondary	1398 (55.9)	1377 (55.1)
Unknown	47 (1.9)	57 (2.3)
Married or stable relationship	2164 (86.5)	2138 (85.5)
No (%) completed weeks of gestation:		
<34	1 (0.04)	0 (0.0)
34-36	201 (8.0)	172 (6.9)
37-40	2123 (84.9)	2158 (86.3)
≥41	176 (7.0)	171 (6.8)
Diabetes mellitus	2 (0.1)	0 (0.0)
Hypertension	54 (2.2)	57 (2.3)
No prenatal care	9 (0.4)	8 (0.3)
Established labour:		
Yes	371 (14.8)	373 (14.9)
No	1140 (45.6)	1098 (43.9)
Uncertain	990 (39.6)	1030 (41.2)
Cervical dilation (cm):		
0	220 (8.8)	215 (8.6)
1	829 (33.2)	809 (32.4)
2	702 (28.1)	670 (26.8)
>2	248 (9.9)	258 (10.3)
Not assessed	502 (20.1)	549 (22.0)
Ruptured membranes	68 (2.7)	58 (2.3)
Previous admission for labour assessment	556 (22.2)	590 (23.6)

Percentages may not sum to 100 owing to rounding or missing data.

Table 2 compares the amount of time spent by the two groups in the labour assessment unit and provides indicators of labour progress before admission to the labour ward. The groups were similar on all indicators.

**Table 2 tbl2:** Indicators of labour progress from randomisation until admission to labour ward. Values are numbers (percentages) unless stated otherwise

Indicator	Structured care (n=2497)	Usual care (n=2499)
Total hours in labour assessment unit after randomisation*:		
Mean (SD)	3.2 (2.5)	3.0 (2.8)
Median (interquartile range)	2.5 (1.5-4.0)	2.1 (1.0-3.9)
No of times discharged undelivered:		
0	1171 (46.9)	1145 (45.8)
1	964 (38.6)	969 (38.8)
2	245 (9.8)	265 (10.6)
>2	112 (4.5)	119 (4.8)†
Stage of labour immediately before transfer to labour and delivery:		
Not established	364 (14.6)	366 (14.7)
Early	1061 (42.5)	1106 (44.3)
Active	953 (38.2)	909 (36.4)
2nd stage	19 (0.8)	16 (0.6)
Unsure	97 (3.9)	101 (4.0)
Cervical dilation immediately before transfer to labour and delivery (cm):		
<3	808 (32.4)	837 (33.5)
3-6	1146 (45.9)	1148 (45.9)
>6	100 (4.0)	91 (3.6)
Not assessed	440 (17.6)	422 (16.9)

*Includes cumulative time for those who had more than one visit.

†A secondary outcome (odds ratio 0.94, 98.75% confidence interval 0.53 to 1.34).

### Outcomes

#### Primary and secondary outcomes

The rate of spontaneous vaginal birth was 64.0% (n=1597) in the structured care group and 61.3% (n=1533) in the usual care group (odds ratio 1.12, 95% confidence interval 0.96 to 1.27; table 3). The groups were comparable for women who had no intrapartum analgesia or anaesthesia and for those requiring suturing for perineal trauma (table 3). Women in the structured care group were less likely to report disappointment with both the amount of attention received from and the helpfulness of care providers in the labour assessment unit (table 4).

**Table 3 tbl3:** Comparisons of maternal outcomes from randomisation until postnatal hospital discharge. Values are numbers (percentages) of women unless stated otherwise

Event	Structured care (n=2497)	Usual care (n=2499)	Odds ratio (CI)
Labour onset:			
Spontaneous	2232 (89.4)	2209 (88.4)	—
Induced	255 (10.2)	283 (11.3)	—
No labour	8 (0.3)	6 (0.2)	—
Oxytocin started after active labour	1553 (62.2)	1587 (63.5)	0.95 (0.77 to 1.12)*
Analgesia or anaesthesia†:			
Regional‡	2112 (84.6)	2159 (86.4)	0.85 (0.62 to 1.08)*
Intramuscular or intravenous opioid	1126 (45.1)	1078 (43.2)	—
Nitrous oxide	167 (6.7)	146 (5.8)	—
Pudendal, paracervical, saddle block	8 (0.3)	4 (0.2)	—
General	16 (0.6)	22 (0.9)	—
Other§	6 (0.2)	2 (0.1)	—
None	112 (4.5)	112 (4.5)	1.06 (0.54 to 1.58)*
Continuous electronic fetal heart rate monitoring	2117 (84.8)	2160 (86.4)	0.84 (0.59 to 1.08)*
Method of delivery:			
Spontaneous vaginal delivery	1597 (64.0)	1533 (61.3)	1.12 (0.96 to 1.27)*
Instrumental vaginal delivery	341 (13.7)	362 (14.5)	—
Vacuum	231	240	—
Forceps (low or mid)	110	122	—
Caesarean delivery	559 (22.4)	604 (24.2)	0.90 (0.71 to 1.10)*
Perineal trauma requiring suturing:	1336 (53.5)	1350 (54.0)	0.98 (0.82 to 1.13)*
Episiotomy	569	573	—
Second degree laceration	764	790	—
Third or fourth degree laceration	131	111	—
Other	3	0	—
Maternal death	1¶	0	—
Health problems during postnatal stay:			
Postnatal fever	24 (1.0)	23 (0.9)	—
Haemorrhage >1000 ml	51 (2.0)	49 (2.0)	—
Transfusion given	13 (0.5)	7 (0.2)	—
Other**	10 (0.4)	4 (0.2)	—
Length of postnatal hospital stay, median (interquartile range), hours	50.1 (41.4, 63.5)	50.3 (41.2, 64.1), P=0.75‡‡	—

*Spontaneous vaginal delivery was primary outcome; prespecified confidence interval 95%. Oxytocin, regional analgesia, electronic fetal heart rate monitoring, and caesarean delivery were “other” outcomes; prespecified confidence interval 99.5%. Perineal trauma requiring suturing was a secondary outcome; prespecified confidence interval 98.75%.

†Some women had more than one form of analgesia or anaesthesia.

‡Epidural analgesia, combined spinal anaesthesia and epidural, or spinal anaesthesia.

§Sterile water injections (n=7) and intrathecal opioid (n=1).

¶Due to undetected haemorrhage from uterine artery after caesarean delivery. Data safety and monitoring committee concluded that death was unrelated to the trial.

**Such as hospital acquired pneumonia; severe pregnancy induced hypertension; septic pelvic thrombophlebitis; severe endometriosis; major delivery complications (tear of small bowel, cystostomy, bladder tear, severe bleeding requiring laparotomy, hysterectomy).

‡‡Prespecified “other” outcome.

**Table 4 tbl4:** Comparisons of women’s reports of negative views of their care.* Values are numbers (percentages)

Item	Structured care (n=2067)	Usual care (n=2064)	Odds ratio (CI)
Helpfulness of nurse or midwife in labour assessment unit:			
Response other than “very helpful”	403 (19.5)	544 (26.4)	0.67 (0.50 to 0.85)†
Attention from staff in labour assessment unit			
Unhappy with amount	233 (11.3)	407 (19.7)	0.51 (0.32 to 0.70)†

*Of 4131 women who completed postnatal questionnaire.

†Secondary outcome; prespecified confidence interval 98.75%.

#### Other immediate maternal outcomes

Comparable numbers of women in both groups were sent home from the labour assessment unit on more than two occasions. In the structured care group 84.6% (n=2112) of women had regional analgesia, compared with 86.4% (n=2159) in the usual care group. The rate of caesarean delivery in the structured care group was 22.4% (n=559), compared with 24.2% (n=604) in the usual care group. One maternal death occurred due to haemorrhage from a uterine artery after caesarean delivery. Other immediate maternal outcomes, including complications and length of postnatal hospital stay, were comparable in both groups.

#### Immediate neonatal outcomes

Because major congenital anomalies influence use of resuscitation and higher level of care as well as length of stay, data from seven babies (three in the structured care group, four in the usual care group) with major congenital anomalies were excluded from analyses of neonatal outcomes (table 5). One stillbirth and no neonatal deaths occurred. Just under 7% of the sample (n=339) required higher level care in a special care baby unit.

**Table 5 tbl5:** Comparisons of immediate neonatal outcomes.* Values are numbers (percentages) unless stated otherwise

Characteristic	Structured care* (n=2494)	Usual care* (n=2495)
Alive at birth	2494 (100)	2494 (99.9)†
Girl	1249 (50.1)	1288 (51.6)
Boy	1245 (49.9)	1207 (48.4)
Mean (SD) birth weight (g)	3394 (431)	3404 (435)
Apgar score <7:		
1 minute	246 (9.9)	234 (9.4)
5 minutes	30 (1.2)	28 (1.1)
Neonatal death	0	0
Higher level of care‡	168 (6.7)	171 (6.9)

*Excludes seven babies (three in structured care group and four in usual care group) born with major congenital anomalies, such as tetralogy of Fallot, brainstem glioma, and transposition of great vessels, that would have affected Apgar scores, resuscitation, level of care, and length of stay.

†Cause of stillbirth was recorded as intrauterine asphyxia.

‡An “other” outcome; odds ratio 0.97 (prespecified confidence interval 99.5% 0.62 to 1.33).

#### Mothers’ and babies’ health 6-8 weeks after discharge

In total 76.0% (n=1570) of mothers in the structured care group rated their general health as excellent or very good compared with 74.7% (n=1542) in the usual care group. One hundred and thirty four (6.5%) mothers in the structured care group scored more than 12 on the Edinburgh postnatal depression scale, compared with 149 (7.2%) in the usual care group (odds ratio 0.84, 99.5% confidence interval 0.36 to 1.32). Most mothers rated their baby’s health as excellent or very good (95.7% in the structured care group, 94.9% in the usual care group). Forty four women in the structured care group and 37 women in the usual care group were readmitted for delivery related complications (odds ratio 1.19, 99.9% confidence interval 0.34 to 2.04). Sixty six babies in the structured care group and 83 in the usual care group were readmitted (0.78, 0.37 to 1.20).

#### Secondary analyses

Completion of some form of post-secondary education and being unmarried were associated with lower probabilities of a spontaneous vaginal birth, whereas being in established labour at trial entry and allocation to structured care were associated with higher probabilities of a spontaneous vaginal birth. In the regression model for main effects the odds ratio (95% confidence intervals) for post-secondary education was 0.82 (0.70 to 0.93), for being unmarried was 0.76 (0.61 to 0.91), for established labour was 1.25 (1.02 to 1.47), and for allocation to structured care was 1.12 (1.00 to 1.26). When interaction terms involving treatment group and education, marital status, and established labour were entered into the model, P values were greater than 0.10.

## Discussion

We evaluated a structured approach to nursing or midwifery care in hospital labour assessment units, which included assessment of and interventions for maternal emotional state, fetal position, and pain, during a minimum of one hour. With the important exception of women’s views of their care (which favoured the structured care group), results did not reach conventional levels of statistical significance. The trend towards increased likelihood of spontaneous vaginal birth suggests that further refinement of structured care is warranted. Although logistic regression analyses found associations between spontaneous vaginal birth and marital status, education, and being in established labour at trial entry, we found no evidence that the between treatment comparisons for spontaneous vaginal birth differed between subgroups defined by these baseline variables.

Compliance was excellent, and reports from the providers of structured care indicated that the intervention was applied appropriately and consistently across and within sites. In this large multicentre trial it would have been prohibitively expensive to directly observe the providers’ actions. We took several measures to prevent contamination. Throughout the trial we emphasised the importance of maintaining distinct study groups and the uncertainty of the value of the experimental approach. Staff providing structured care were volunteers who were favourably disposed towards the type of care. Staffing was such that usual care rarely allowed for one to one attention for 1-4 hours, as required in structured care. As with our previous trial of another complex labour intervention in which blinding was impossible,^23^ we have indirect evidence that treatment fidelity was maintained. Women’s evaluations of the helpfulness of and amount of attention received from staff were more favourable in the structured care group (table 4). Furthermore the hospital that enrolled the largest number of women to the trial (970) also had the highest risk of contamination; it was a self contained unit staffed by two nurses on each shift and yet its odds ratio for spontaneous vaginal birth was statistically significant (1.34, 95% confidence interval 1.03 to 1.75).

Complex interventions such as structured care have the advantage of mirroring the real world of practice, in which assessments and interventions are tailored to individual needs. Furthermore, synergistic effects among components of an intervention would be lost if each were evaluated individually.^30^ However, complex interventions have the disadvantage of leaving some uncertainty about the importance of each component of the intervention.^31^ Our approach reflected current best practice guidelines, by addressing context, collecting the best evidence, developing a conceptual model to explain the links between intervention components and outcome, and standardising the intervention.^30^ ^32^ None the less, the labour assessment units did not seem to delay admission to the labour ward, as nearly 60% of participants (n=2897) were not in active labour when admitted (table 2).

Two early trials on labour have addressed the question of location of care.^33^ ^34^ One trial enrolled 209 women in one hospital in Ontario and randomised them to either a labour assessment unit or direct admission to the labour ward.^34^ The other trial enrolled 1459 women in seven hospitals in British Columbia (Canada) and randomised them to care either at home or in hospital.^33^ We evaluated an approach to the content (rather than the place) of care. Given the low intensity of the intervention, the absence of evidence of risk, the potential population effects if it were adopted, the continuing rise in caesarean delivery rates, and the beneficial effect on satisfaction with care, hospitals that already have labour assessment units may want to consider incorporating structured care into routine practice.

A combination of structured care plus strict adherence to a policy of delayed admission to the labour ward until clinically indicated may yield greater benefits. Questions remain about the optimum setting for women in latent phase labour and about the characteristics of hospitals that influence the effectiveness of forms of intrapartum nursing or midwifery care. Further research is warranted, given the paucity of effective interventions to increase the likelihood of spontaneous vaginal birth in healthy childbearing women.

**What is already known on this topic**

Prolonged latent phase labour is associated with increased risk of operative delivery and neonatal morbidity
Hospital labour assessment units are prevalent in North America but uncommon elsewhere

**What this study adds**

A formalised approach to care in a hospital labour assessment unit improves women’s views of their care and may increase the likelihood of spontaneous vaginal birth
Labour assessment units may want to consider standardising the care provided to include assessment and interventions for maternal psychological state, pain, and positioning

## Notes

We thank the participants, staff at the participating hospitals, and the structured care trainers (Melrose East, Adele Hood, Debbie Kaye, Debbie Patrick, Anne Simmonds, Ann Sprague, and Marie-Josée Trépanier); members of the Data Safety Monitoring Committee—Rona McCandlish (chair), Shoo Lee, and David Young; members of the Structured Early Labour Assessment and Care by Nurses Trial Group who enrolled participants and collected data—Paula Baker, Amy Hailey, Charlotte Ice, Karen Langston, and Gina Smith (Harris Methodist Fort Worth Hospital, Fort Worth, TX), Nancy Fehr, Lynn Hickey, and Maggie Quance (Foothills Medical Centre, Calgary, AB), Susan Caffrey, Cheryl Hardy, Maggie Hickey, Carol Luppi, and Ginny Silva (Brigham and Women’s Hospital, Boston, MA), Judy Claringbold, Jill Fairbairn, Linda McCabe, and Janice McVeety (Ottawa Hospital—Civic Campus, Ottawa, ON), Heather Brown, Jackie Clark, Una Dewtie, Ruth Jordan, Patsy Smith, and Erna Snelgrove (IWK Health Centre, Halifax, NS), Gerry Ashton, Isabelle Baribeau, Janet Britnell, Janet Brownlee, Brenda Cook, Isabelle Gagnon, Adriana Hoselton, Nicole Joly, Debbie Kaye, Ann Salvador, and Julia Watson-Blasioli (Ottawa Hospital—General Campus, Ottawa, ON), Anne Fowler, Lucy Gilmore, Althea Stewart, Debbie Walker, and Linda Young (William Osler Health Centre, Brampton, ON), Luisa Ciofani, Helen Doulos, Francine Martin, Beatrice Stoklas, and Jennifer Supino (McGill University Health Centre, Montreal, QC), Jeannine Alt, Mary Knapp, Sandy Lynne, Denice O’Connor, and Becky VanLaan (Spectrum Health, Grand Rapids, MI), Tania Hansen, Judy Jones, and Teresa Stanfill (St Luke’s Regional Medical Center, Boise, ID), Chris Jabaay, Carol Lawrence, Nancy Travis, Kim Vincent (Lee Memorial Health System, Fort Myers, FL), Gaylene Kramchynski, Susan Mussell, and Kris Robinson (St Boniface General Hospital, Winnipeg, MB), Annette McHugh, Glenys Roberts, Louise Taylor, and Grace Thomas (Royal Gwent Hospital, Gwent Healthcare NHS Trust, Newport, Wales), Pat Bielecki, Laurie Lang, Tammi Lothson, Debbie Lunardini, and Robyn Magnuson (Advocate Health Care—Lutheran General Hospital, Park Ridge, IL), Hazel Canavan, Tina Davis-Larkin, Elaine Perkins, Sue Riegel, and Lorian Williams (Advocate Health Care—Christ Hospital, Oak Lawn, IL), Carole England, Jayne Gregory, Debbie Patrick, and Hora Soltani (Derby City General, Derby, UK), Linda Koehl, Marcia Patterson, and Cheryl Stone (Rush University Medical Center, Chicago, IL), Patti Janssen, Monica Nicol, Lynne Palmer, and Lesley Smith (Surrey Memorial Hospital, Surrey, BC), and Carol Burke, Joy Grohar, Abby Hornbogen, and Donna Pemberton (Northwestern Memorial Hospital, Chicago, IL).

Contributors: All authors participated in writing the original grant application, were members of the steering committee to manage the trial, and contributed to all drafts of the manuscript. EH was the principal investigator and led the trial team. She contributed to analysing and interpreting the data, wrote the first draft, and is guarantor. AW was the trial statistician and contributed to the data analysis plan and supervision and is guarantor for the analyses. JW was the trial coordinator. During the time of the trial NKL was with the School of Nursing, Oregon Health and Science University, Portland, OR, USA.

Ethical approval: Research ethics committees at the University of Toronto and by all participating hospitals.

Provenance and peer review: Not commissioned; externally peer reviewed.

## Notes

**Cite this as:** *BMJ* 2008;337:a1021
